# C-X-C Motif Chemokine Ligand 14 is a Unique Multifunctional Regulator of Tumor Progression

**DOI:** 10.3390/ijms20081872

**Published:** 2019-04-16

**Authors:** Xiao-Yan Yang, Shigeyuki Ozawa, Yasumasa Kato, Yojiro Maehata, Kazuhito Izukuri, Takeharu Ikoma, Keisuke Kanamori, Tetsu Akasaka, Kenji Suzuki, Hiroshi Iwabuchi, Shun-Ichi Kurata, Iyoko Katoh, Takashi Sakurai, Tohru Kiyono, Ryu-Ichiro Hata

**Affiliations:** 1 Oral Health Science Research Center, Graduate School of Kanagawa Dental University, Yokosuka 238-8580, Japan; yang@kdu.ac.jp (X.-Y.Y.); ozawa@kdu.ac.jp (S.O.); maehata@kdu.ac.jp (Y.M.); izukuri@kdu.ac.jp (K.I.); t.ikoma@kdu.ac.jp (T.I.); mmafighter5050@gmail.com (K.K.); akasaka@kdu.ac.jp (T.A.); kurata@kdu.ac.jp (S.-I.K.); katoh.iyoko@kdu.ac.jp (I.K.); 2 Department of Dentomaxillofacial Diagnosis and Treatment, Graduate School of Kanagawa Dental University, Yokosuka 238-8580, Japan; k.suzuki@kdu.ac.jp (K.S.); iwabuchi@kdu.ac.jp (H.I.); t.sakurai@kdu.ac.jp (T.S.); 3 Nippi Research Institute of Biomatrix, 520-11 Kuwabara, Toride, Ibaraki 302-0017, Japan; 4 Department of Oral Function and Molecular Biology, Ohu University School of Dentistry, Koriyama 963-8611, Japan; y-katou@den.ohu-u.ac.jp; 5 Department of Oral Science, Graduate School of Kanagawa Dental University, Yokosuka 238-8580, Japan; 6 Department of Critical Care Medicine and Dentistry, Graduate School of Kanagawa Dental University, Yokosuka 238-8580, Japan; 7 Division of Carcinogenesis and Cancer Prevention, Department of Cell Culture Technology, National Cancer Center Research Institute, Tokyo 104-0045, Japan; tkiyono@ncc.go.jp

**Keywords:** C-X-C motif chemokine ligand 14, multifunctional tumor suppressor, antimicrobial function, molecular preventive medicine

## Abstract

Cancer is a leading cause of death and disease worldwide, with a tremendous financial impact. Thus, the development of cost-effective novel approaches for suppressing tumor growth and progression is essential. In an attempt to identify the mechanisms responsible for tumor suppression, we screened for molecules downregulated in a cancer progression model and found that the chemokine CXCL14, also called BRAK, was the most significantly downregulated. Increasing the production of CXCL14 protein by transfecting tumor cells with a CXCL14 expression vector and transplanting the cells into the back skin of immunodeficient mice suppressed tumor cell growth compared with that of parental tumor cells, suggesting that CXCL14 suppressed tumor growth in vivo. However, some studies have reported that over-expression of CXCL14, especially in stromal cells, stimulated the progression of tumor formation. Transgenic mice expressing 10-fold more CXCL14 protein than wild-type C57BL/6 mice showed reduced rates of chemical carcinogenesis, transplanted tumor growth, and metastasis without apparent side effects. CXCL14 also acts as an antimicrobial molecule. In this review, we highlight recent studies involving the identification and characterization of CXCL14 in cancer progression and discuss the reasons for the context-dependent effects of CXCL14 on tumor formation.

## 1. Introduction

Chemokines (chemotactic cytokines) are a group of structurally-related proteins with molecular sizes ranging from 8–12 kDa that regulate the trafficking of various types of leukocytes by interacting with a subset of G protein-coupled receptors. Each chemokine is named according to the arrangement of the N-terminal cysteine residues within it. The two major subfamilies, defined by the presence of four conserved cysteine residues linked by two disulfide bonds, are the CC and CXC chemokines. They are distinguished according to the position of the first two-cysteine residues, which are adjacent to each other (CC subfamily) or separated by one amino acid (CXC subfamily). In the tumor microenvironment, chemokine expression determines the distribution of immune cells and thus controls the overall immune response to the tumor, thereby playing an integral role in the regulation of cancer progression and metastasis [1,2,3].

In this report, we review recent studies on the identification and characterization of CXC ligand 14 (CXCL14) in cancer progression and provide an analysis of future perspectives in this research field.

## 2. Identification of CXCL14 as an Endogenous Tumor Growth Suppressor

### Screening for Tumor-Suppressor Candidates

Tumors develop via a multistep process [4,5], and tumor progression is dependent on the balance of the expression between tumor progression-promoting and -suppressing genes [6].

To identify promising target molecules for applications in the treatment of head and neck squamous cell carcinoma (HNSCC), we employed tongue carcinoma-derived HSC-3 cells in culture. Epidermal growth factor (EGF) signaling is frequently overactivated in various carcinoma cells, including HNSCC cells; thus, we cultured HSC-3 cells under serum-free conditions, treated them with EGF to mimic tumor progression in vivo, and identified genes whose expression was significantly altered using DNA chip analysis followed by quantitative real-time polymerase chain reaction [7]. Expression levels of mRNAs for matrix metalloproteinase-1 (*MMP-1*), also known as animal collagenase, and vimentin were significantly increased. In contrast, the expression levels of the tissue inhibitor of matrix metalloproteinase 3 (*TIMP3*), which inhibits the activity of various MMPs, and insulin-like growth factor (IGF) binding protein-3 (IGFBP-3), which suppresses the activities of IGF-1 and-2 by binding to these growth factors with high affinity, were significantly down-regulated. However, the down-regulation of the chemokine CXCL14 [8] was the most prominent [7].

Many investigators have screened molecules that are overexpressed during tumor progression as target molecules for therapeutic drugs and have attempted to prevent tumor progression by inhibiting their activity or suppressing the expression levels of these tumor-promoting molecules, such as MMP-1. However, because these molecules are usually essential for normal development, drugs inhibiting these target molecules are often not successful in clinical applications owing to their serious side effects. In contrast, activation of presumptive tumor suppressors or inhibition of the down-regulation of such targets may be a much more promising approach for the prevention of tumor progression without severe side effects, because these molecules are sometimes present in normal tissues.

CXCL14, also called BRAK because it was initially found to be expressed in the breast and kidneys [8], is ubiquitously and abundantly expressed in almost all cell types, particularly epithelial cells, and was found to be significantly down-regulated in our model system. Thus, our data suggested that CXCL14 could act as a tumor suppressor.

The rate of tumor formation in vivo in CXCL14-expressing vector-transfected tumor cells (HSC-3-CXCL14) in athymic nude mice or T and B cell-deficient SCID mice was significantly lower than that of mock vector-transfected control cells, although the growth rates of the expression vector-transfected and mock vector-transfected cells were not different under in vitro culture conditions [7,9,10]. In addition, the volume of tumors formed in vivo by CXCL14-expressing cells was significantly smaller than that of mock-transfected cells [7,9,10,11]. To clarify whether expression of CXCL14 affected the settlement of carcinoma cell clones in host tissues in vivo and/or proliferation of the colonized carcinoma cells, we engineered oral floor carcinoma-derived HSC-2 cells in which CXCL14 expression was inducible upon doxycycline treatment. The cells were treated with doxycycline before xenografting, and the host mice were pretreated with the drug or the mice were inoculated with untreated HSC-2 cells and then treated with the drug after the establishment of tumor xenografts in the host; tumor sizes were subsequently measured. The tumor xenografts were significantly smaller in mice treated with doxycycline than in control untreated mice, irrespective of whether doxycycline treatment was performed before or after xenografting, even though the growth rate of the engineered cells was the same in the presence or absence of doxycycline in vitro. Immunohistochemical studies showed that maturation of blood vessels in the tumors was suppressed by treatment with doxycycline. Thus, these data indicated that CXCL14 expression in oral floor carcinoma cells reduced both the rate of settlement and the proliferation of the cells in vivo after the settlement of the cells [11]. Moreover, CXCL14 expression was reduced in tongue carcinoma tissue compared with that in surrounding normal tissues [12], indicating that CXCL14 exhibits tumor-suppressive activity toward HNSCC in vivo [13].

## 3. CXCL14 Expression as a Marker for Suppression of Tumors by Cetuximab, an Anti-EGF Receptor (EGFR) Monoclonal Antibody

Cetuximab (Erbitux), a human-murine chimeric monoclonal antibody directed to the EGFR ligand-binding site, has been successfully used to treat some patients with colorectal cancer and HNSCC. For effective treatment, it is essential to first identify cetuximab-responsive patients. The level of EGFR expression and the presence of mutations in signaling molecules downstream of the EGFR pathway have been reported to be determining factors for cetuximab responsiveness in patients with colorectal cancer; however, limited data have been reported for patients with HNSCC. In our previous work, we employed HSC-3 cells with low levels of CXCL14-expression and CXCL14-nonexpressing YCU-H891 cells as representatives of these two groups and compared their responsiveness to cetuximab and the expression of CXCL14 under various conditions. The growth of xenografted tumors derived from HSC-3 cells expressing CXCL14 in vivo and in vitro under serum-free culture conditions was suppressed by injection of cetuximab into tumor-bearing mice; however, neither the expression of the chemokine nor the cetuximab-dependent suppression of xenografted tumor growth was observed for YCU-H891 cells [14]. Both types of cells expressed wild-type EGFR and did not harbor mutations in downstream signaling molecules that have been reported to be mutated in patients with cetuximab-resistant colon cancer [14]. We found that the CXCL14 promoter region in YCU-H891 cells was hypermethylated and that demethylation of the promoter by treatment with 5-aza-2′-deoxycytidine (DAC) restored CXCL14 mRNA expression and in vivo cetuximab-mediated tumor growth suppression. Finally, we observed in vivo tumor growth suppression when YCU-H891 cells were engineered to express CXCL14 ectopically in the presence of doxycycline [14]. These results indicated that CXCL14 expression could be a good predictive biomarker for cetuximab-dependent tumor suppression and that evaluation of CXCL14 expression could be useful for discrimination of cetuximab-susceptible and -resistant carcinomas, thereby benefiting patients by potentially reducing treatment time and costs.

## 4. Expression of CXCL14 in Carcinoma Cells and Growth of Tumors

Our data indicated that CXCL14 expression in carcinoma cells suppresses tumor growth in vivo by acting in an autocrine or paracrine fashion [7,9,10,11]. Downregulation of CXCL14 associated with carcinoma progression was later reported for various other types of carcinomas, including adenocarcinomas and squamous cell carcinomas [15,16,17,18]. However, increased expression of CXCL14 has also been reported in adenocarcinomas [19,20,21,22]. For example, CXCL14 mRNA levels are significantly up-regulated in localized prostate cancer, and CXCL14 expression levels are positively correlated with Gleason scores in prostate cancer [19]. In spite of these data, overexpression of CXCL14 in prostate cancer (LAPC4) cells by introducing mouse or human CXCL14 expression vectors suppresses tumor growth in vivo compared with the growth of control vector-transfected tumor cells [19]. These data demonstrated that the apparent increase in CXCL14 expression in carcinomas does not support the in vivo effects of CXCL14 upregulation on tumor growth. One possible explanation of this apparent discrepancy between CXCL14 expression and tumor growth is that although the expression of CXCL14 is stimulated in tumor cells, its tumor inhibitory activity may not be sufficient to suppress tumor growth. Alternatively, the presence of other molecules in addition to CXCL14 may also affect tumor growth.

## 5. CXCL14 Produced by Carcinoma-Associated Fibroblasts (CAFs) and Growth of Tumors

Recent in vivo and in vitro studies have demonstrated that the growth and invasive behaviors of carcinomas are influenced by surrounding stromal cells, including fibroblasts, myofibroblasts, leukocytes, and myoepithelial cells [23,24].

Molecular characterization of the tumor microenvironment in breast cancer has shown that dramatic changes in gene expression occur in tumor-associated stromal cells and cancerous epithelial cells. The chemokines CXCL14 and CXCL12 are overexpressed in tumor myoepithelial cells and myofibroblasts, respectively, as demonstrated in a study using serial analysis of gene expression [25]. To investigate the functions of CXCL14 in prostate tumor stromal cells, Augsten et al. produced CXCL14-secreting NIH-3T3 (NIH-CXCL14) cells by introducing the human CXCL14 cDNA sequence into mouse NIH-3T3 cells [26]. NIH-CXCL14 cells promoted the growth of prostate cancer cell xenografts and increased tumor angiogenesis. Further comparison of CXCL14 expression in the normal and tumor stroma revealed a statistically significant increase in stromal expression of CXCL14 in 15 of 27 (56%) cancer samples; thus, the authors claimed that CXCL14 promoted in vivo growth [26].

There are at least three possible explanations for the apparent discrepancy in the effects of CXCL14 on tumor progression. First, CXCL14 may have cell type-specific functions. Second, the presence of other factors in addition to CXCL14 may alter its functional effects. The third possibility is that CXCL14 may also stimulate multiple signals and induce contrasting effects such as tumor suppression or tumor progression.

In terms of the first explanation, to investigate whether CXCL14 produced by mesenchymal-derived cells shows tumor-progressive effects, we employed mesenchyme-derived fibrosarcoma cells (MC57) and produced a stable cell line expressing CXCL14 (MC57-CXCL14) or control mock vector (MC57-MOCK). Transplanting MC57-CXCL14 cells into the back skin of C57BL/6 mice resulted in the formation of smaller tumors compared with those transplanted with control MC57-MOCK cells, suggesting that CXCL14 expression in mesenchyme-derived cells also suppresses tumor growth. Ras-homologous small GTPase (RhoA) and Rho-associated coiled-coil-containing protein kinase (ROCK) are important regulators of secretory processes. Thus, we investigated the effects of fasudil, a specific inhibitor of ROCK, on CXCL14 secretion and tumor progression in these cells. Fasudil significantly increased CXCL14 secretion by MC57-CXCL14 cells in a concentration-dependent manner. To determine the effects of fasudil on tumor growth, MC57-CXCL14 and MC57-MOCK cells were transplanted into wild-type C57BL/6 mice. Fasudil treatment suppressed tumor growth only in mice that had received MC57-CXCL14 cell transplants. These results indicated that fasudil inhibited fibrosarcoma growth by stimulating CXCL14 secretion [27]. These data suggest that both epithelial cell- and mesenchymal cell-derived CXCL14, when working in an autocrine manner, has tumor-suppressive effects in a transplanted tumor system.

Second, the presence of other factors in addition to CXCL14 may also alter its functional effects. CXCL14 is not an angiogenic molecule, but has been characterized as angiostatic [28,29]. NIH-CXCL14 cells, but not recombinant CXCL14, enhance the in vitro proliferation and migration of prostate cancer cells and promote in vivo angiogenesis in the NIH-CXCL14 system [26]. This finding suggests that the effects of CXCL14 observed above could be related to the presence of additional factors, such as CXCL12, because CXCL12 has tumorigenic and angiogenic effects under some conditions [30]. Recent reports have shown that CXCL14 can bind to CXCR4, a receptor for CXCL12, resulting in modulation of the activity of CXCL14 [31,32]. In particular, when present at high concentrations (300 nM), i.e., 3000 times higher than blood levels of CXCL14 in wild-type mice or the mean blood concentration in healthy humans [33] and 300 times higher than that in the transgenic mice described above, CXCL14 binds to CXCR4 and modulates the three-dimensional structure of the receptor to stimulate the binding and activity of CXCL12 [34]. NIH-3T3 cells are often used in various studies because they can easily incorporate foreign genes and express high levels of recombinant gene products. Data using these cells have suggested that the high concentration of CXCL14 in NIH-CXCL14 cells and co-presence of molecule(s) such as CXCL12 could explain the protumorigenic and angiogenic effects of NIH-CXCL14 cells [26,32,34].

The third possibility has been reported in glioblastoma (GB) cases [35]. GB is the most common and deadliest malignant primary brain tumor with a high recurrence rate. The long non-coding(lnc) RNA UCA1/micro RNA (miR)-182 axis has been regarded as a nodal driver of glioma invasion mediated by GB-associated stromal cells (GASCs) and GASC-secreted chemokine CXCL14. In clinical specimens, CXCL14 upregulation in GASCs also correlated with poor prognosis. Notably, CXCL14-high GASCs mediated lncRNA UCA1 upregulation and miR-182 downregulation in glioma cells. Moreover, miR-182 directly binds to fructose-2,6-biphosphatase; thus, the UCA1/miR-182 axis thereby modulates GASC-induced glycolysis in glioma cells. Overall, the UCA1/miR-182/PFKFB2 axis is associated with chemokine CXCL14 secretion, glycolysis, and invasion of glioma cells in GASCs [35]. Stimulation of glycolysis and the resulting extracellular acidity due to an increase in lactate concentration, a process known as “aerobic glycolysis” or the “Warburg Effect”, is a hallmark of solid tumors [36]. Interestingly, acidic extracellular pH has been found to increase intracellular Ca^2+^ and matrix metalloproteinase-9 expression, as well as increasing experimental lung metastasis of melanoma cells [37]. These data suggest that CXCL14 could have bidirectional functions that are context-dependent.

GPR85, a candidate receptor for CXCL14, was found on the cell membranes of primary mammary fibroblasts obtained from benign breast mass (normal-associated fibroblasts, NAFs). CXCL14 bound to GPR85 and activated the conversion of NAFs into CAFs. The chemokine CCL17, secreted by CAFs, promoted the epithelial-mesenchymal transition and lung metastasis of breast cancer cells [38]. Thus, GPR85 may be a good target molecule for further clarification of context-dependent CXCL14 functions on tumor progression.

## 6. CXCL14 is a Multifunctional Tumor Suppressor

To further investigate whether carcinoma-associated cells suppressed the growth of tumor cells other than HNSCC cells in a paracrine or endocrine manner, we produced CXCL14-overexpressing transgenic mice and examined the growth of tumor cell transplants in these mice. We prepared transgenic mice by injecting beta-actin promoter-regulated human CXCL14 cDNA into the pronuclei of fertilized eggs for male C57BL/6J mice to achieve CXCL14 expression in various cell types, including epithelial and stromal cells. We established and used three independent transgenic mouse lines to determine the actual effects of the incorporated transgene, and not dependent on the destruction of a putative tumor-promoting gene that might be present in parental mice. The serum levels of CXCL14 protein in the three lines were 8, 11, and 14 ng/mL, which were approximately 10 times higher than that in wild-type C57BL/6 mice (0.9 ng/mL). These transgenic mice showed reduced growth of Lewis lung carcinoma (LLC) cell and B16 melanoma cell transplants, indicating that CXCL14, which was first identified as a suppressor of tumor progression when overexpressed in HNSCC cells also suppressed adenocarcinoma derived from LLC cells and melanoma cells [29]. These cell lines were chosen in part because they did not produce their own CXCL14; therefore, any effects could be expected to be a result of the overexpressed transgene in the microenvironment acting in a paracrine or endocrine manner [29].

Significant growth suppression of LLC tumor cell transplants was observed in our three independent lines of CXCL14 transgenic mice [29], indicating that tumor suppression was due to the high expression of CXCL14 in the transgenic mice and not to the destruction of any putative tumor progression stimulator, which may have been produced during the establishment of the transgenic lines. Moreover, we observed less number of blood vessels penetrated into transplanted tumors in transgenic mice compared with that in wild-type mice, further supporting the anti-angiogenic effects of CXCL14 [29].

Cancer progression involves carcinogenesis, an increase in tumor size, and metastasis; these processes are dependent on the mutations or abnormal activation of different molecules. Using the transgenic mice described above, we found that the rate of azoxymethane/dextran sodium sulfate-induced colorectal carcinogenesis in these mice was significantly lower than that in isogenic wild-type mice [39]. The growth of transplanted B16 melanoma cells and LLC cells was also suppressed in transgenic mice compared with that in wild-type mice, indicating that CXCL14 suppressed both carcinogenesis and tumor growth [39]. When mice were treated with NK cell-depleting antibody (anti-asialo GM1 antibody), growth rates of tumors were much faster in both Wt and Tg mice, even though in these conditions, tumor sizes were much smaller in Tg mice.

To find the actual effects of CXCL14 on metastasis in transgenic mice, we employed an experimental metastasis system and found that for both melanoma cells and LLC cells, the number of nodules in the lungs of the transgenic mice was significantly lower than that observed for the wild-type animals [39]. In this case, also NK cell-depleting antibody (anti-asialo GM1 antibody) reduced the suppression rate of metastasis in Tg mice. Many suppressors of individual processes have been reported; however, no reports have described molecules other than CXCL14 that suppress all the tumor progression steps. Treatment of Wt and Tg mice with α-galactosyl ceramide, which stimulates NKT cell function, synergistically suppressed metastasis of melanoma cells with CXCL14. These data suggest that both NKT and NK cells are important for CXCL14 Tg mice to show the suppressive effect of tumor growth and metastasis.

## 7. Increased Survival Rates after Injection of Melanoma Cells into CXCL14 Transgenic Mice

To investigate the effects of overexpression of *CXCL14* on the life span of mice, we used the Kaplan-Meir method to determine the survival rates after injection of various numbers of B16 melanoma cells. The rate of survival was always significantly higher in transgenic mice than in wild-type mice, indicating that high expression of CXCL14 increased the survival rate and decreased tumor cell metastasis. The three CXCL14 transgenic founders were crossed with isogenic wild-type C57BL/6 mice, and the birth rates of males and females were determined for each line. There were no significant differences between the distribution of sex and transgenic genes [39], suggesting that a 10-fold increase in the expression of CXCL14 in the blood was tolerable and did not affect birth or development. The presence of an individual expressing this level of CXCL14 in the blood in the healthy population [33] also supported the idea that this level of high expression does not cause severe side effects in humans. Further studies are needed to investigate the relationship between the levels of blood CXCL14 and cancer incidence.

## 8. Antimicrobial Function

**Figure 1:**
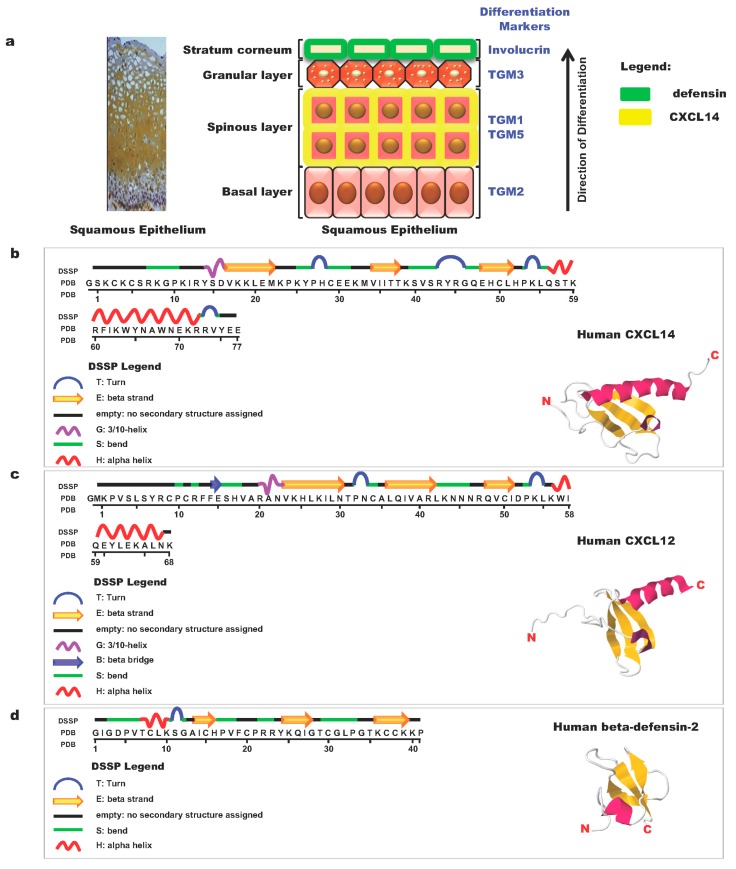
Schematic illustration of the structure of skin epithelial cell layers and representative molecules expressed in these layers [42]. (**a**) The structure of the squamous epithelial cell layer and localization of differentiation marker proteins, chemokines, and defensin. In the left panel, the tissue was treated with anti-human CXC ligand 14 (CXCL14) antibodies (brown in color). TGM: Transglutaminase. (**b**) Primary and three-dimensional structures of the human chemokine CXCL14 and its domain structure [43]. (**c**) Primary and three-dimensional structures of the human chemokine CXCL12 and its domain structure [44]. (**d**) Primary and three-dimensional structures of human beta-defensin-2 and its domain structure [45]. Structures of molecules were adopted from respective references.

The skin is constantly exposed to commensal microflora and pathogenic microbes. The skin is composed of layers of keratinocytes at different stages of differentiation. CXCL14 is not found in the cornified layer of the outermost skin but is expressed mainly in the spinous cell layer (Figure 1a) [40]. The basic molecular structure of CXCL14 is shown in Figure 1b, together with those of CXCL12 (Figure 1c) and human beta-defensin-2 (Figure 1d). Beta-defensin-2 (Figure 1d) is a typical antimicrobial peptide (AMP) and is localized in the outer-most layer of the squamous epithelium. CXCL14 is an AMP with broad-spectrum activity and the ability to kill cutaneous gram-positive bacteria and *Candida albicans* as well as the gram-negative enterobacterium, *Escherichia coli*. This novel AMP is abundantly expressed in the dermis and epidermis of healthy human skin but is downregulated under conditions of inflammation and disease, suggesting that CXCL14 fights bacteria at the earliest stage of infection [40,41] and may indirectly suppress cancer initiation by preventing infection-induced inflammation.

## 9. Regulation of *CXCL14* Expression

### 9.1. The Mitogen-Activated Protein Kinase (MAPK)/Extracellular Signal Regulated Kinase (ERK)/p38 Signaling Pathway Regulates CXCL14 Expression

**Figure 2:**
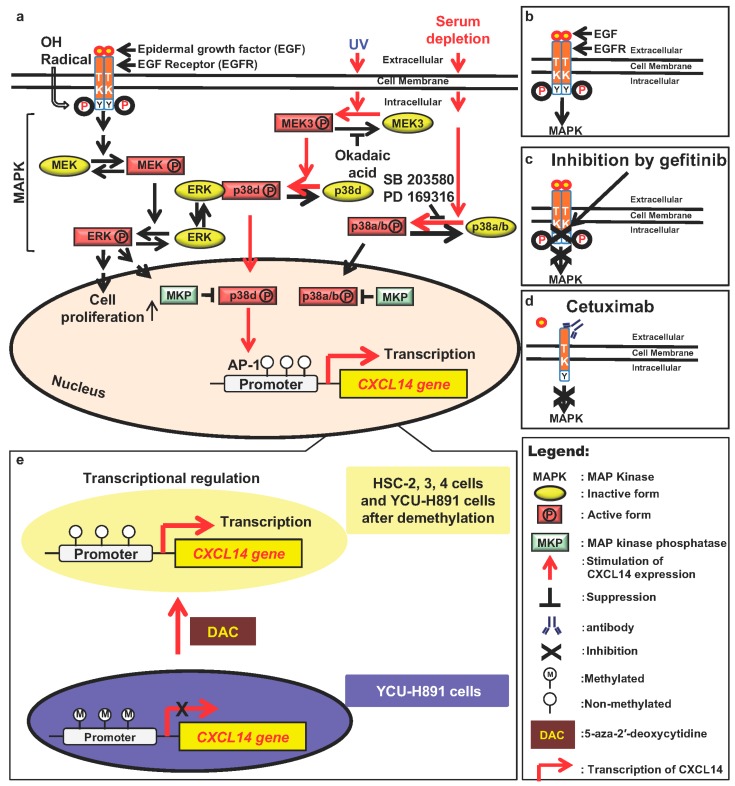
Regulation of CXCL14 expression by mitogen-activated protein kinase (MAPKs) and EGF receptor (EGFR) activators/inhibitors. Red arrows indicate signals that stimulate CXCL14 expression, while black T-bars and crosses indicate suppression of CXCL14 expression. Molecules in yellow ellipses are inactive forms, whereas those molecules in red rectangles are active forms. (**a**) Regulation of CXCL14 expression by extracellular signals, ERK, and p38 MAPK. (**b**) EGF binding to EGFR and stimulates MAPK activity [7,9,46]. (**c**). Gefitinib binds to EGFR and suppresses MAPK stimulation [10]. (**d**) Cetuximab binds to EGFR and suppresses MAPK activity [14]. (**e**) DAC suppresses cytidine methylation and stimulates the transcription of CXCL14 [14].

An increase in the number of cell-surface EGFR molecules and overactivation of EGFR and its downstream signaling pathways due to mutations induce overgrowth of cells in vivo and in vitro (Figure 2a,b). Using various inhibitors, we showed that the EGFR/MEK/ERK pathway indeed down-regulates CXCL14 mRNA expression (Figure 2a) [10]. Next, we examined whether modulation of CXCL14 mRNA expression by EGF and/or gefitinib, an inhibitor of EGFR, is reflected in protein levels of CXCL14 and whether gefitinib treatment attenuates the EGF effect by elevating the CXCL14 protein level. Western blot analysis clearly demonstrated that EGF induced CXCL14 repression and that gefitinib treatment restored CXCL14 expression at the protein level (Figure 2b,c) [10]. Cetuximab, a monoclonal antibody that specifically binds to EGFR, also suppresses MAPK/ERK and stimulates the expression of CXCL14 (Figure 2d) [14]. The MAPK family includes ERK, c-Jun N-terminal kinase, p38, and ERK5 (big-MAPK, BMK1). We also showed that the stress-dependent effects of p38 isoforms are responsible for the upregulation of CXCL14 expression (Figure 2a) [46]. Thus, the discovery or development of an edible small molecule that stimulates CXCL14 expression in the human body may be a useful and cost-effective method of cancer prevention.

### 9.2. Transcriptional Regulation of CXCL14

To study the regulatory mechanisms governing the expression of this gene, we determined the transcriptional start site and promoter motifs of the gene. The major transcriptional start site determined by use of the 5′ rapid amplification of cDNA ends was found to be located in the first proposed exon 1 region (+284) of the gene. Determination of the luciferase activities of reporter gene constructs with various deletions or mutations showed that an atypical TATA-like sequence, TATTAA, was essential for the transcription of the gene and that the AP-1 binding sequence and tandem GC box were necessary for stimulating the expression of the gene in human squamous epithelial cells. The DNA region was highly homologous (95% base identity) to the mouse gene. In addition, okadaic acid, an inhibitor of serine/threonine phosphatases 1 and 2A, stimulated TATTAA sequence- and AP-1 binding sequence-dependent promoter activity and increased the level of CXCL14 mRNA, indicating that these sequences are essential for regulation of CXCL14 (Figure 2a) [47].

### 9.3. Epigenetic Regulation of CXCL14 Expression

When cultured HNSCC cells were treated with cetuximab, a monoclonal antibody against EGFR or gefitinib, a specific inhibitor of EGFR, *CXCL14* expression was increased in HSC-2, HSC-3, and HSC-4 cells, but not in YCU-H891 cells. HSC-2, HSC-3, and HSC-4 cells transplanted into the backs of immune-defective nude mice grew well, and the growth of these xenograft tumors was suppressed by treatment with gefitinib and/or cetuximab [10,14]. In contrast, the growth of YCU-H891 cell transplants was not suppressed by cetuximab treatment. Cancer develops via mutations in growth-regulating genes. However, mutations in such genes, which have been reported to occur in colon cancer, were not found in either HSC-3 or YCU-H891 cells [14]. In addition to gene mutations, epigenetic changes in genes due to modification by methylation of the promoter sequence of the genes are often found in cancer cells. In fact, we could detect methylation in 50% of the cytidine residues in the promoter region in YCU-H891 cells, but not in HSC-3 cells [14]. Moreover, the addition of 5-aza-2′-deoxycytidine (DAC), an inhibitor of cytidine methylation to the culture medium of YCU-H891 cells in addition to cetuximab stimulated the expression of CXCL14 (Figure 2e). Additionally, injection of DAC together with cetuximab in YCU-H891 tumor-bearing mice stimulated CXCL14 expression in the tumor and suppressed tumor growth, indicating that CXCL14 was a marker for tumor suppression by cetuximab [14].

### 9.4. Mechanisms of Tumor Suppression by CXCL14

The specific molecular mechanisms through which CXCL14 suppresses tumor growth are unclear. A recent study reported that CXCL14 acts as a specific carrier of CpG DNA into dendritic cells and activates Toll-like receptor 9-mediated adaptive immunity [48]. This is an interesting mechanism that could explain the suppression of carcinogenesis, tumor growth, and metastasis. Collagen-induced arthritis (CIA) is an animal model for the human autoimmune disease rheumatoid arthritis. CIA was induced by prior dermal injection of chicken type II (cartilage type) collagen in complete Freund’s adjuvant. CXCL14-overexpressing transgenic mice exhibit exacerbated CIA symptoms [49], suggesting that these mice are much more sensitive to foreign antigens than wild type C57BL/6 animals. These data are consistent with the idea that CXCL14 suppresses various types of tumors in transgenic mice.

## 10. Conclusions

In transgenic mice overexpressing CXCL14, the rate of chemical carcinogenesis, the growth of transplanted tumors, and experimental metastasis of carcinoma cells were all suppressed. CXCL14 also showed antimicrobial effects against various types of bacteria, including gram-negative and gram-positive bacteria, suggesting that CXCL14 is a multistep and multifunctional self-defense molecule. The homologous primary structure of the protein and functional characteristics of the mouse and human molecules suggest that CXCL14 may function similarly in humans. The high expression of CXCL14 in blood in healthy humans and transgenic mice did not appear to have any negative side effects, suggesting that this molecule may be a good target for the development of cost-effective methods for cancer therapy and prevention.

The apparent contradictory or context-dependent effects of CXCL14 on tumor formation have been discussed. Further studies are needed to clarify the specific molecular mechanisms and/or effect of levels of CXCL14 on tumor suppression and regulation of CXCL14 gene expression in order to facilitate the application of CXCL14 as a molecular target in cancer therapy and prevention.

## Abbreviations

CXCL14	C-X-C motif chemokine ligand 14
DAC	5-Aza-2′-deoxycytidine
HNSCC	Head and neck squamous cell carcinoma
EGF	Epidermal growth factor
MMP-1	Matrix metalloproteinase-1
TIMP3	Matrix metalloproteinase 3
IGF	Insulin-like growth factor
IGFBP-3	Insulin-like growth factor binding protein-3
NIH-CXCL14	CXCL14-secreting NIH-3T3
CAFs	Carcinoma-associated fibroblasts
NAFs	Normal-associated fibroblasts
MC57	Mesenchyme-derived fibrosarcoma cells
RhoA	Ras-homologous small GTPase
ROCK	Rho-associated coiled-coil-containing protein kinase
LLC	Lewis lung carcinoma
AMP	Antimicrobial peptide
TGM	Transglutaminase
